# Loss of MTX2 causes mitochondrial dysfunction, podocyte injury, nephrotic proteinuria and glomerulopathy in mice and patients

**DOI:** 10.7150/ijbs.89916

**Published:** 2024-01-12

**Authors:** Ting Li, Ying Bao, Yu Xia, Hanyan Meng, Chao Zhou, Limin Huang, Xiaowen Wang, En Yin Lai, Pingping Jiang, Jianhua Mao

**Affiliations:** 1 Department of Nephrology, The Children's Hospital of Zhejiang University School of Medicine, National Clinical Research Center for Child Health, National Children's Regional Medical Center, Hangzhou, China.; 2 Department of Pediatric Nephrology, Xi'an Children's Hospital, The Affiliated Children's Hospital of Xi'an Jiaotong University, Xi'an, China.; 3 Department of Pediatric Nephrology, Wuhan Children's Hospital (Wuhan Maternal and Child Healthcare Hospital), Tongji Medical College, Huazhong University of Science & Technology, Wuhan, China.; 4 Kidney Disease Center of the First Affiliated Hospital and Department of Physiology, School of Basic Medical Sciences, Zhejiang University School of Medicine, Hangzhou, China.; 5 Institute of Pharmaceutical Biotechnology, Zhejiang University School of Medicine, Hangzhou, China.

**Keywords:** MTX2, proteinuria, glomerulopathy, podocyte, mitochondrial dysfunction

## Abstract

Proteinuria is a common and important clinical manifestation of chronic kidney disease (CKD) and an independent risk factor for the progression of kidney disease. As a component of the glomerular filtration barrier (GFB), podocyte plays a key role in the pathogenesis of glomerular diseases and proteinuria. However, the pathophysiology of glomerular diseases associated with mitochondrial function is incompletely understood. Here, we identified three novel mutations in* MTX2*, encoding a membrane protein in mitochondria, associated with multisystem manifestations including nephrotic proteinuria and kidney injury in two Chinese patients. Conditional podocyte-specific Mtx2 knockout (Pod-Mtx2-KO) mice present a series of podocyte and glomerular abnormalities from 8 weeks to old age, including microalbuminuria, glomerular mesangial hyperplasia, fusion and effacement of foot process. MTX2 deficiency impaired podocyte functions *in vitro,* manifested by reductions of adhesion, migration and endocytosis, which were further restored by overexpression of MTX2. Moreover, MTX2 defects led to abnormal mitochondrial structure and dysfunction, evidenced with defects of complex I and III, increased production of reactive oxygen species (ROS), and decreased protein levels of Sam50-CHCHD3-Mitofilin axis in the mitochondrial intermembrane space bridging (MIB) complex which is responsible for maintaining mitochondrial cristae morphology. Collectively, these findings reveal that the normal expression of MTX2 in glomerulus plays an important role in the adhesion, migration, endocytosis, proliferation and other physiological functions of podocytes, which may be realized by maintaining the morphological structure and function of mitochondria. Abnormal expression of MTX2 can lead to mitochondrial dysfunction and structural abnormalities by Sam50-CHCHD3-Mitofilin axis in podocyte, which further induces podocyte injury, glomerular lesions and proteinuria.

## Introduction

Chronic kidney disease (CKD) is an important risk factor for end-stage kidney disease, cardiovascular and cerebrovascular events and all-cause death 1. Proteinuria is a common and important clinical manifestation of CKD and an independent risk factor for the progression of kidney disease 2. The glomerular filtration barrier (GFB) is composed of endothelial cells, glomerular basement membranes (GBM), podocytes and slit diaphragm (SD), which is the last barrier to prevent the occurrence of proteinuria. The integrity of podocytes is essential for maintaining the normal structure and function of GFB. Thereby, the irreversible damage or loss of podocytes is a critical pathological basis for massive proteinuria and glomerulosclerosis 3.

Mitochondrial cytopathies caused by mitochondrial DNA (mtDNA) or nuclear DNA (nDNA) mutations can lead to podocyte injury and glomerulopathy. The well-known glomerular diseases associated with mitochondrial cytopathies are mitochondrial myopathy, encephalopathy, lactic acidosis, strokes (MELAS) syndrome caused by mtDNA 3243A>G mutation 4,5 and coenzyme Q10 biosynthesis defect caused by mutation of nuclear gene encoding coenzyme Q10 6-9, manifesting in proteinuria and focal segmental glomerulosclerosis (FSGS) in adults and children. Podocytes as highly differentiated cells are particularly energy-dependent. Oxidative phosphorylation injury in podocytes leads to increased reactive oxygen species (ROS) production, causing mitochondrial dysfunction and structural abnormalities in podocytes, resulting in the destruction of the GFB and the occurrence of proteinuria, which may progressively develop into glomerulosclerosis 10,11.

Metaxin 2 (MTX2) is a mitochondrial outer membrane protein composed of 263 amino acids that is widely expressed in various tissues (Uniprot: O75431). The gene encoding MTX2 contains 12 exons on 2q31.1 and is localized to the cytoplasmic side of the mitochondrial outer membrane by binding to its partner Metaxin1 (MTX1) 12. The Sorting and Assembly Machinery (SAM) complex in the mitochondrial outer membrane consists of three core components: MTX1, MTX2 and Sam50 13, which helps the β-barrel proteins to fold correctly and assemble into the mitochondrial outer membrane 14,15. The SAM complex and the mitochondrial contact site and cristae organizing system (MICOS) complex located in the inner mitochondrial membrane together constitute the core components of the mitochondrial intermembrane space bridging (MIB) complex, which is associated with the maintenance and regulation of mitochondrial cristae 16,17. In addition, MTX1 and MTX2 are involved in TNF- α induced apoptosis 18-21.

Elouej et al. 22 first reported five *MTX2* homozygous mutations in seven children with mandibuloacral dysplasia progeroid syndrome (MDPS; OMIM#619127), including growth retardation, bone resorption, arterial calcification, severe hypertension and renal glomerulosclerosis. However, the mechanisms by which MTX2 deficiency results in kidney injury have not been reported and elucidated. Here, we identified three novel *MTX2* mutations associated with multisystem manifestations including proteinuria and glomerulosclerosis in two Chinese child. Along with detailed clinical evaluations, we investigated the role of MTX2 deficiency in podocyte injury and proteinuria by generating conditional podocyte-specific Mtx2 knockout (Pod-Mtx2-KO) mice.

## Results

### Clinical manifestations in patients with *MTX2* mutations

**Figure 1:**
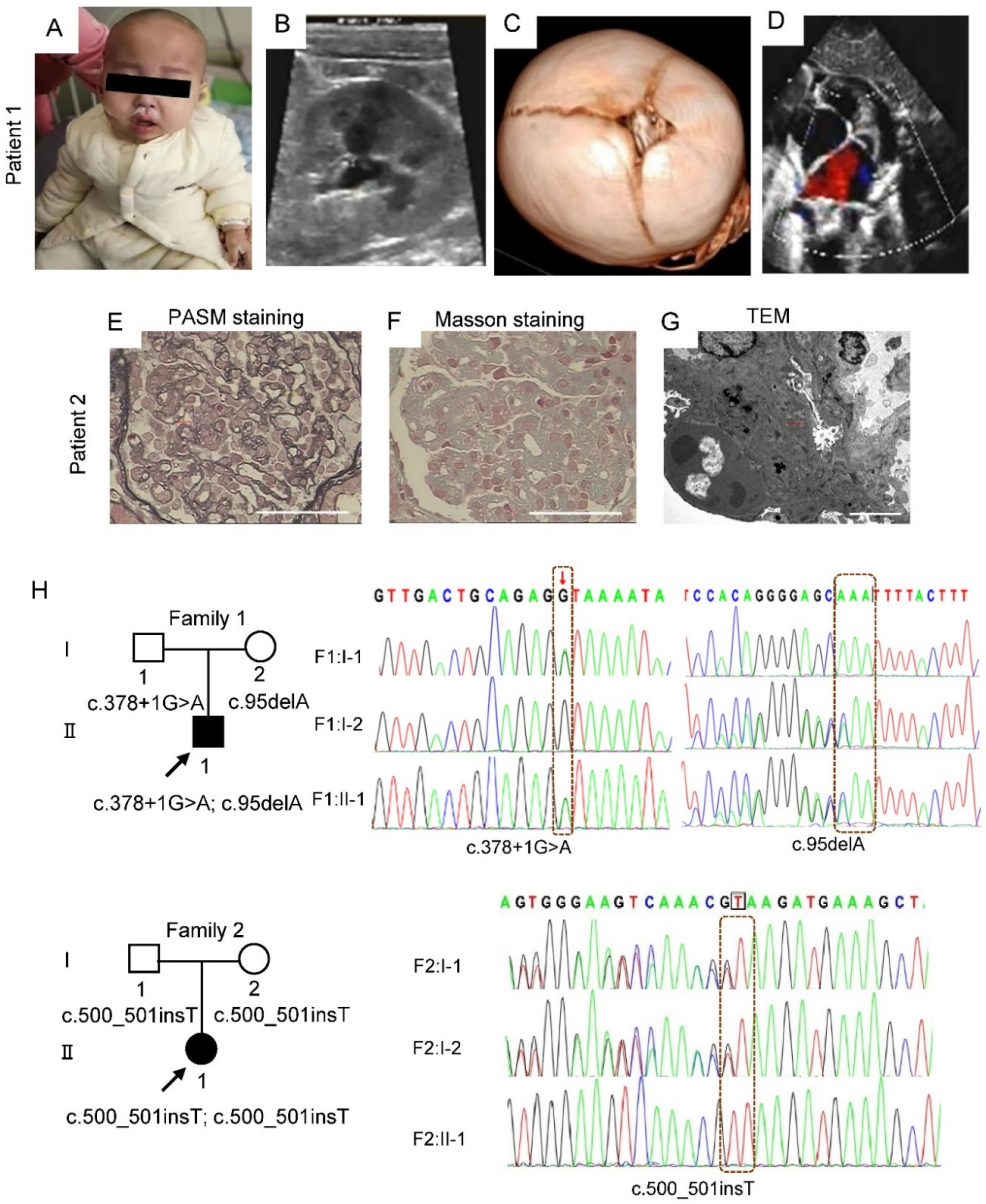
** Clinical manifestations and mutations of gene *MTX2* in probands. (A)** Photograph of patient 1. The boy showed specific facial features (sparse hair; frontal bossing; exophthalmos; prominent ear; thick lip; crowding teeth; micrognathia).** (B)** Renal ultrasound of patient 1.** (C)** Cranial CT in patient 1.** (D)** Echocardiography of patient 1.** (E)** Representative images of PASM staining in kidney of patient 2. yellow arrows, “pseudo double track”, red arrows, thickening of basement membrane, scale bar: 25 μm.** (F)** Masson staining in kidney of patient 2. red arrows, deposition of fuchsinophilic protein in mesangial area and basement membrane, scale bar: 25 μm.** (G)** Transmission EM images in kidney of patient 2. red arrows, electronic dense deposits in subendothelial, mesangial areas and subepithelial, scale bar: 5 μm.** (H)** *MTX2* mutations in families. The compound heterozygous mutation including c.378+1 (IVS6) G>A mutation and c.95 (exon3) delA mutation in patient 1 and the c.500 (exon8) _c.501 (exon8) insT homozygous mutation in patient 2 was identified by WES. Circles indicate females; squares indicate males; solid indicate patients; arrows indicate patients.

Patient 1, a boy aged 1 year and 11 months from Wuhan in China, presented with nephrotic-range proteinuria (24-hour urine protein 333.33 mg/kg), facial edema, oliguria, hypertension (141 - 155 mmHg/85-124 mmHg), and pneumonia. Physical examination showed growth retardation (height 73 cm, < 3rd; weight 9 kg < 3rd), paucity of speech, muscular hypotonia, difficulty in standing and walking, specific facial features (Figure 1A). Laboratory tests showed elevated level of plasma lactate (4.64 mmol/l), anemia (Hb 96 g/l), abnormal liver function (ALT 1064 U/l) and myocardial injury (CK-MB 104 U/l; LDH-L 1310 U/l; NT-proBNP 7071 pg/ml). Renal ultrasound demonstrated fluid dark areas (anteroposterior diameter 0.7 cm) in bilateral renal pelvis and enhanced echoes in duplex renal parenchyma (Figure 1B). Renal dynamic imaging identified mildly decreased blood perfusion and glomerular filtration in the right kidney (glomerular filtration rates, 40.54 ml/min and 26.17 ml/min in left and right kidney, respectively), and bilateral upper urinary tract excretory obstruction (Figure S1). Cranial computerized tomography (CT) showed unclosed bregma, widened cranial suture, slightly enlarged bilateral lateral ventricles and wide cerebral sulcus (Figure 1C). Echocardiography demonstrated atrial septal defect (secondary hole, sieve-like) and pulmonary hypertension (Figure 1D).

Patient 2, a girl aged 4 years and 2 months from Xian province of China, presented with steroid resistant nephrotic syndrome (24-hour urine protein 110.68 mg/kg, serum albumin 18.5 g/l), microscopic hematuria, edema of face and limbs, hypertension (140-170 mmHg/100-120 mmHg), hyperspasmia, bilateral hydronephrosis of renal pelvis, enlarged liver and spleen, abdominal distension, vomiting, headache, reversible posterior encephalopathy syndrome and pneumonia. Physical examination showed reduced binocular vision, ascites, hepatosplenomegaly and lymphadenopathy, myodynamia IV-V, hypomyotonia, positive left-sided Babinski sign, and specific facial features (Cushingoid appearance; orbital hypertelorism; micrognathia). Laboratory examinations identified increased level of plasma lactate (6.50 mmol/l), glomerular hematuria (urinary red blood cells 40-50 cells/HP, deformability 100%) and hypoalbuminemia (serum albumin 18.5 g/l). Renal biopsy revealed atypical membranoproliferative nephropathy in Xi'an children's Hospital (August 16, 2018) (Figure 1E-G).

By whole exome sequencing (WES) and Sanger sequencing, compound heterozygotes c.95delA (p. Q32Qfs*26) and c.378 + 1G > A were identified in patient 1 and a novel homozygous mutation c.500_501insT (p. R167Rfs*2) in patient 2 in the *MTX2* gene (NM_006554), whose segregation pattern fit an autosomal recessive model of inheritance (Figure 1H). The two-frameshift mutations, c.95delA and c.500_501insT, are absent in GnomAD database and result in a truncating protein either. The splicing mutation c.378+1G > A is recorded with an allele frequency of 0.0000133 in GnomAD. Based on ACMG guidelines 23, three mutations were classified as pathogenic or likely pathogenic with prediction of damage effects on protein function. However, parents were healthy as carriers with heterozygote, and parents in family 2 were consanguineous marriage carrying the same insertion.

### Proteinuria, glomerulopathy and podocyte injury in Pod-Mtx2-KO mice

**Figure 2:**
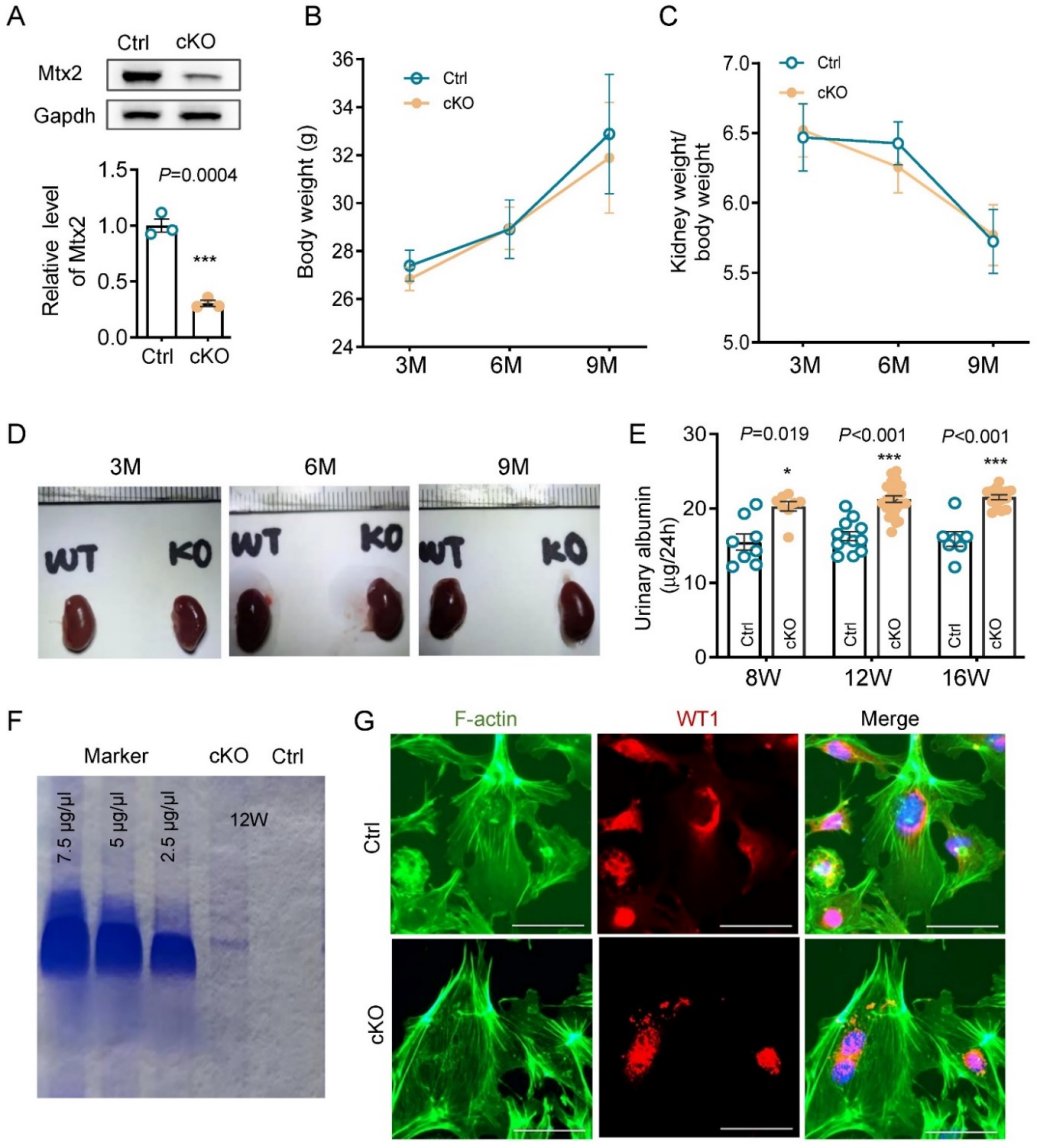
** Renal phenotype in Pod-Mtx2-KO mice. (A)** Western blots and quantification analysis, showing reduced MTX2 expression in primary glomerular cells from Pod-Mtx2-KO mice compared with control mice (n = 3).** (B)** The summary data on body weights of mice (n = 10).** (C)** The summary data on kidney weights of mice (n = 10).** (D)** Representative images of kidney from Pod-Mtx2-KO and control mice at 3, 6, and 9 mo of age.** (E)** 24 h urinary albumin quantification of Pod-Mtx2-KO and control mice at 8, 12, 16 weeks of age (n = 10).** (F)** Coomassie blue staining of urine from Pod-Mtx2-KO and control mice at 12 weeks of age as well as standard BSA with different concentrations.** (G)** Representative images showing F-actin cytoskeleton stained with FITC labeled phalloidin in primary podocytes isolated from Pod-Mtx2-KO and control mice at 8 weeks of age, scale bars: 50 μm. WT1, a specific marker protein of podocytes to identify isolated primary glomerular podocytes. Data was shown as the mean ± SD of triplicates at least. *, *P* < 0.05, **, *P* < 0.01, ***, *P* < 0.001.

Podocytes play a key role in development of proteinuria and glomerulosclerosis. By immunohistochemistry and immunofluorescence, it is clear that MTX2 is rich in podocytes, mesangial cells in glomeruli (Figure S2). Then we used Cre-Loxp system based on podocin-Cre to construct conditional Pod-Mtx2-KO mice (Figure S3). The level of Mtx2 in primary glomerular cells from Pod-Mtx2-KO mice (Figure S4) was substantially detected by western blotting, approximately 30% of the controls (Figure 2A). There were few differences in mice body weight, kidney weight or kidney size observed between Pod-Mtx2-KO and WT mice at 3, 6, and 9 months of age (Figure 2B-D). Microalbuminuria was initially occurred at 8 weeks and distinctly developed at 12 weeks with 21.22 μg/24 h of urinary albumin compared to 16.24 μg/24 h in WT mice (Figure 2E and Table S1). Urinary albumin at 12 weeks was further confirmed by Coomassie blue staining (Figure 2F). However, no significant difference in the expression of actin fiber of cytoskeleton architecture was exhibited in primary podocytes between Pod-Mtx2-KO and WT mice at 8 weeks (Figure 2G).

**Figure 3:**
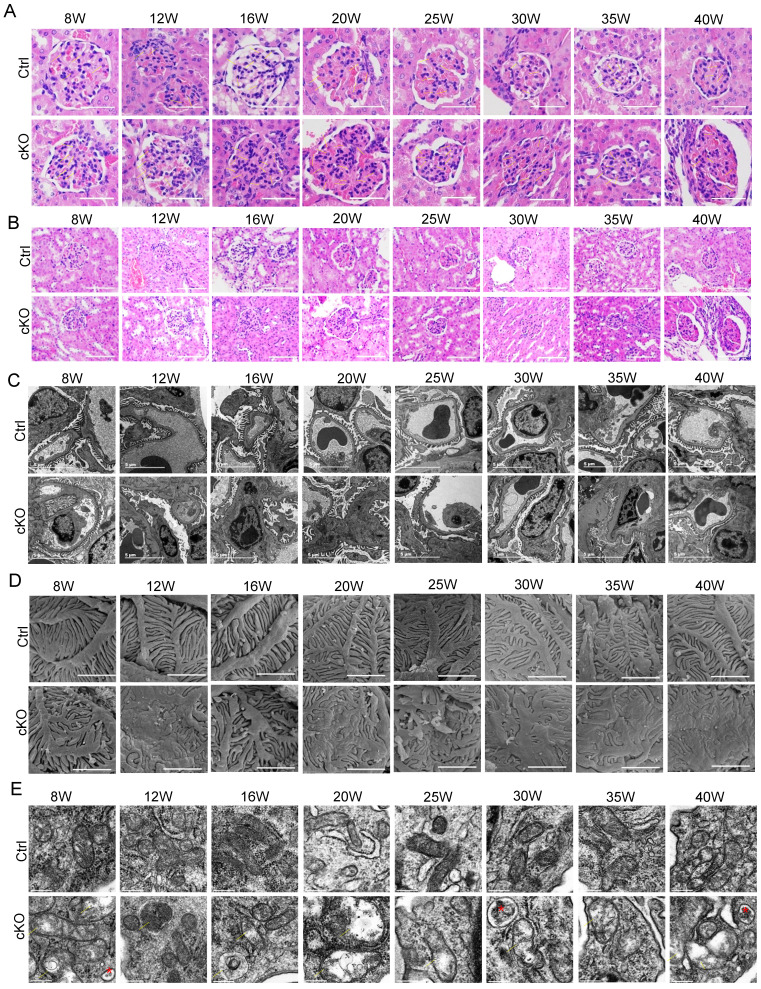
** Renal histology and ultrastructural morphology in Pod-Mtx2-KO mice. (A)** HE staining of glomerular mesangial hyperplasia in different degrees in Pod-Mtx2-KO mice kidneys during the 40 week follow-up (n = 6), yellow arrows, mesangial cells, scale bar: 50 μm.** (B)** Images of HE staining demonstrating no significant difference in renal tubules between Pod-Mtx2-KO and control mice during the 40 week follow-up (n = 6), scale bar: 100 μm.** (C)** Transmission EM images, showing fusion of podocyte foot processes, abnormal basement membrane and mesangial matrix proliferation in glomerulus of Pod-Mtx2-KO mice at different ages (n = 6), scale bar: 5 μm.** (D)** Images of scanning EM showing abnormal podocyte foot process architecture in Pod-Mtx2-KO mice at different ages (n = 6), scale bar: 2 μm.** (E)** Images of transmission EM illustrating mitochondrial structural abnormalities and increased autophagosomes in podocytes of Pod-Mtx2-KO mice during the 40 week follow-up (n = 6), yellow arrows, abnormal structure of mitochondria; red asterisk, autophagosomes, scale bar: 250 nm.

To well understand the development of renal failure in Pod-Mtx2-KO mice, we took a 40 weeks' follow-up. The histological examination of Pod-Mtx2-KO mice kidneys by HE staining demonstrated different degrees of glomerular mesangial hyperplasia and normal features of renal tubules, but the degree of glomerular mesangial hyperplasia did not show significant regularity with the increase of weeks (Figure 3A, B). Transmission electron microscopy (TEM) and scanning EM were performed to gain an insight into the ultrastructural changes in the glomeruli. In Pod-Mtx2-KO mice from 8 weeks of age, prominent fusion of partial podocyte foot processes, loss of subpodocytic space, bare or thickened basement membrane and hyperplastic mesangial matrix proliferation in glomerulus could be observed by TEM (Figure 3C). Consistent with these results, abnormal podocyte foot process architecture (disorganized arrangement; deformation; fusion), structural alterations of SD and exposed basement membrane were identified in Pod-Mtx2-KO mice from 8 weeks of age by scanning EM (Figure 3D). With the increase of weeks, there was no obvious regularity in the extent of glomerular and podocyte injury observed by TEM and scanning EM. Together, these results indicated that deficiency of Mtx2 led to podocyte injury and subsequently proteinuria in mice. Furthermore, abnormal mitochondrial structure (vacuolated and swelling mitochondria; cristae fragmentation) and increased autophagosomes were visualized by TEM in the podocytes of Pod-Mtx2-KO mice (Figure 3E), suggesting the involvement of mitochondria due to the deficiency of Mtx2 in podocyte.

### MTX2 is required for the maintain of podocyte functions

**Figure 4:**
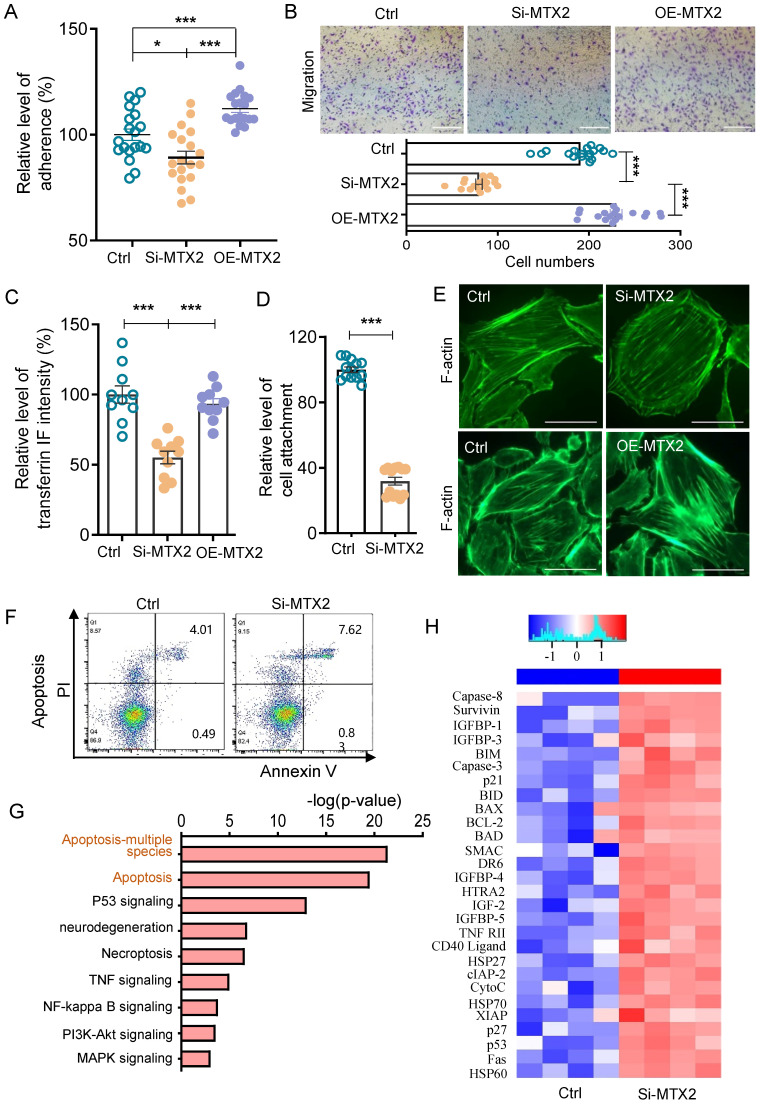
** Podocyte function in MPC5 with or without MTX2 and apoptosis protein microarray assay. (A)** Quantification of adherent podocytes after podocytes were seeded on plates coated with collagen for 1 h.** (B)** Representative images and quantification of migrating podocytes after migration through transwell chamber membranes for 24 h, scale bar: 250 μm.** (C)** Quantification of the mean fluorescence intensity of FITC-transferrin engulfed by podocytes for 5 h.** (D)** Quantification of attached podocytes with or without puromycin aminonucleoside (PAN) treatment.** (E)** F-actin cytoskeleton stained with FITC labeled phalloidin in podocytes, scale bar: 50 μm.** (F)** Podocyte apoptosis measured by propidium iodide (PI) and FITC-conjugated Annexin V.** (G)** Representative images of Kyoto Encyclopedia of Genes and Genomes (KEGG) pathway analysis by mouse apoptosis protein microarray performed on MTX2-KD and control podocytes.** (H)** Representative heatmap of mouse apoptosis associated proteins by mouse apoptosis protein microarray. Data was shown as the mean ± SD of triplicates at least. *, *P* < 0.05, **, *P* < 0.01, ***, *P* < 0.001.

To further examine the influence of MTX2 on podocyte, we generated the conditionally immortalized mouse podocyte cell line (MPC5) with MTX2 knockdown (MTX2-KD) or overexpression by lentiviral transfection *in vitro* (Figure S5). As shown in Figure S5, MTX2 was localized with mitochondria. Considering podocytes morphology and function are vital for the develop of proteinuria and glomerulosclerosis 3, we evaluated its adhesion, migration and endocytosis ability. As illustrated in Figure 4A-C, deficiency of MTX2 notably reduced the adhesion, migration, and endocytosis abilities of podocyte to 89% (*P* < 0.05), 40% (*P* < 0.001) and 55% (*P* < 0.001) that of the controls, respectively. Furthermore, these reductions were restored by overexpression of exogenous MTX2, indicating that MTX2 was required for maintain of those podocyte functions. Consistently, loss of MTX2 led to increased abscission of podocytes with treatment of PAN that only 32% (*P* < 0.001) podocytes retained stuck compared with the controls (Figure 4D). However, no significant alterations were found in actin fiber of cytoskeleton architecture either MTX2 knockdown or overexpression (Figure 4E). A little bit increase of apoptosis was observed in podocytes with abolished MTX2 (Figure 4F). To investigate genes mostly involved in podocyte apoptosis induced by MTX2 deficiency, podocytes from MTX2-KD and control groups were collected for apoptotic protein microarray assay. A total of 38 apoptotic indicators were examined, and protein levels of 28 factors were found to be increased, among which the protein levels of pro apoptotic factors including BIM, BAX, BAD, Capase-3, Capase-8, and CytoC etc. were significantly up-regulated, along with upregulated apoptosis inhibitors, such as BCL2, XIAP and cIAP-2 (Figure 4G, H and Table S2). Thus, our data here demonstrated that the MTX2 deficiency impaired podocyte function.

### MTX2 deficiency impairs mitochondrial function

**Figure 5:**
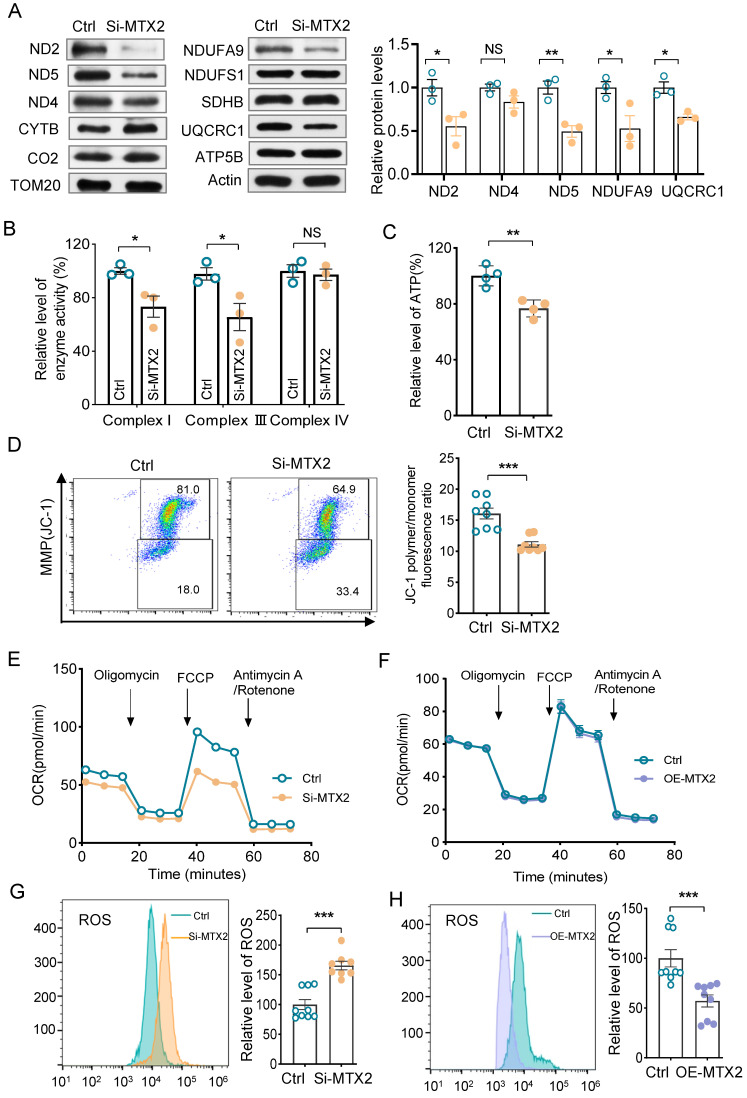
** Mitochondrial function in MPC5 with or without MTX2. (A)** Western blots and quantifications analysis of some subunits in respiratory chain complex. TOM20 as the loading control.** (B)** Quantifications of enzyme activities of oxidative respiratory chains (complex I, III and IV) in MTX2-KD and control podocytes.** (C)** Quantifications of whole cell ATP production in MPC5 with MTX2 knockdown.** (D)** Measurement of MMP using the fluorescence probe JC-1 assay kit in MTX2-KD and control podocytes.** (E)** The OCR curves of podocytes with and without MTX2, stimulated with different inhibitors (oligomycin (1.5 μM), FCCP (2 μM), Retenone /Antimycin (0.5 μM)) at specific time points and quantification of the OCRs.** (F)** The OCR curves of podocytes with MTX2 overexpressing, compared with the control.** (G)** Mitochondrial ROS assayed by flow cytometry using the fluorescent probe DCFH-DA in MTX2-KD and control podocytes.** (H)** Mitochondrial ROS measurement with MTX2 overexpression, compared to the controls. Data was shown as the mean ± SD of triplicates at least. *, *P* < 0.05, **, *P* < 0.01, ***, *P* < 0.001.

Vacuolated mitochondria were present remarkably in podocytes from Pod-Mtx2-KO mice, indicating disturbance of mitochondrial function (Figure 3E). To determine whether the MTX2 deficiency lead to mitochondrial dysfunction, we first measured the protein levels of respiratory chain complex subunits, encoded by mtDNA and nDNA, in MTX2-KD and control cells. As shown in Figure 5A, the levels of subunits ND2, ND5 (encoded by mtDNA) in complex I and subunits NUDFA9 in complex I and UQCRC1 in complex III (encoded by nDNA) in MTX2-KD cells were decreased significantly to 55%, 50%, 53% and 66% of that of the controls. Whereas the levels of subunits SDHB in complex II, CO2 in complex IV, and ATP5B in complex V were not significantly changed. Defects of subunits usually resulted in impaired activity of the respiratory chain complex. As expected, the enzyme activities of complex I and complex III were reduced to 73.2% (*P* < 0.05) and 60.1% (*P* < 0.05) of that of the controls (Figure 5B). Strikingly, altered activities were absent in complex II, IV and V (Figure 5B, Figure S6A). Moreover, overexpression of MTX2 had few effects on activity of complex II or IV (Figure S6B), implying that the defects of complex I and complex III were the main cascade effects from MTX2 deficiency. In addition, reduced ATP production and mitochondrial membrane potential (MMP) were observed in MTX2-KD podocytes, with mean values of 76.7% (*P* < 0.01) and 69% (*P* < 0.001), respectively, compared with the control group (Figure 5C, D). Furthermore, the oxygen consumption rate (OCR) was measured by the XF-96 Extracellular Flux Analyzer to estimate mitochondrial oxidative phosphorylation activity. The OCR curve was declined in MTX2-KD podocytes (Figure 5E), in line with the respiratory complex dysfunction, thereby affecting the energy metabolism of podocytes. However, cells with enriched exogenous MTX2 had similar OCR curve with the control group (Figure 5F). Intriguingly, additional 65.7% (*P* < 0.001) ROS production was accumulated in MTX2-KD podocytes compared to the control group (Figure 5G), and overexpression MTX2 eliminated approximately half ROS that of controls (Figure 5H). Whereas, there were no significantly changes in ATP or MMP in podocytes with MTX2 overexpressing (Figure S6C, D). Together, these data evidenced that MTX2 deficiency led to mitochondrial dysfunction, especially defects of complex I and III, which were the major contributors for ROS generation.

### MTX2 defects alter Sam50-CHCHD3-Mitofilin axis

**Figure 6:**
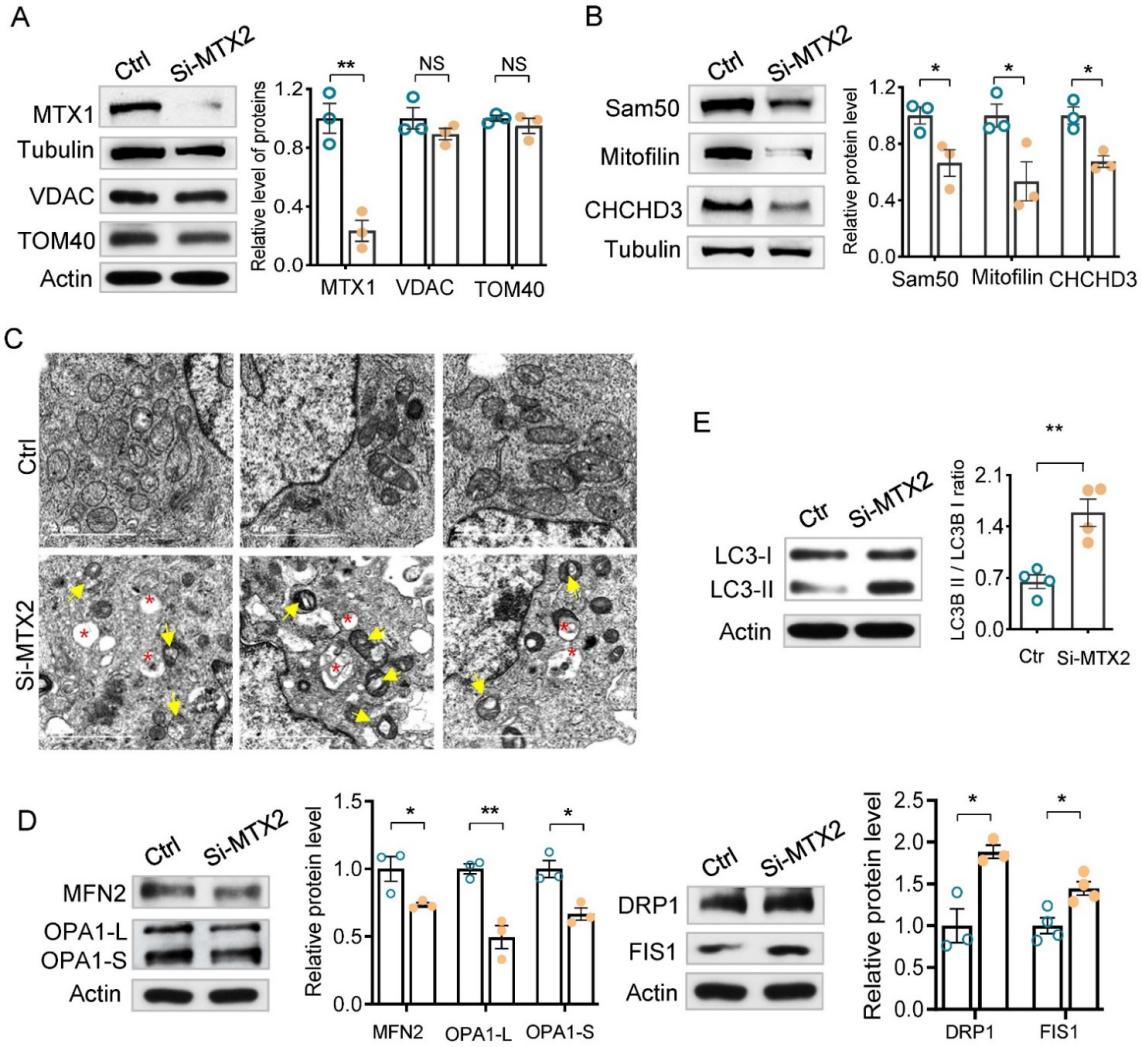
** Alterations in mitochondrial dynamics, structure, membrane proteins and the Sam50-CHCHD3-Mitofilin axis. (A)** Western blots and quantification analysis of mitochondrial membrane proteins (MTX1, VDAC, TOM40) in MTX2-KD and control podocytes.** (B)** Western blots and quantification analysis of the Sam50-CHCHD3-Mitofilin axis in MTX2-KD and control podocytes.** (C)** Images of mitochondrial ultrastructure by transmission EM in MTX2-KD podocytes. yellow arrows, abnormal structure of mitochondria; red asterisk, autophagosomes, scale bars: 2 μm.** (D)** Western blots assay in MTX2-KD podocytes compared to controls, observing decreased levels of mitochondrial fusion proteins MFN2 and OPA1 (including OPA1-L and OPA1-S) and increased levels of mitochondrial fission proteins DRP1 and FIS1.** (E)** Western blots and quantification analysis showing increased LC3-II/I ratio in MTX2-KD podocytes. Data was shown as the mean ± SD of triplicates at least. *, *P* < 0.05, **, *P* < 0.01, ***, *P* < 0.001.

Mitochondrial homeostasis is a prerequisite for maintaining mitochondrial function thus controlling cell fate. Considering MTX2, MTX1 and Sam50 together compose the SAM complex at the mitochondrial outer membrane and vacuolated mitochondria in Pod-Mtx2-KO mice, we inferred that the defects of MTX2 directly disturbed the SAM complex, which in turn regulated the mitochondrial cristae morphology resulting in mitochondrial dysfunction. As shown in Figure 6A, part of MTX1 was lost, with a mean level of 23.5% (*P* < 0.01) relative to the control, due to MTX2 deficiency as described previously 22. However, few changes were detected in the levels of Tom40 and VDAC1/2, which are major components of the SAM complex. As Sam50 interacts with Mitofilin and CHCHD3 in MIB complex that accounts for the maintenance of mitochondrial cristae structures, we thereby assessed the level of Sam50, CHCHD3 and Mitofilin in MTX2-KO cells. As expected, they were significantly decreased to a mean level of 66.4% (*P* < 0.05), 53.4% (*P* < 0.05), and 67.3% (*P* < 0.05) compared with the controls, respectively (Figure 6B). Subsequently, swollen and vacuolated mitochondria, fragmented and disordered cristae and even concentric cristae were identified in MTX2-KD podocytes (Figure 6C), the same as those observed in Pod-Mtx2-KO mice, illustrating that MTX2 deficiency was responsible for abnormal mitochondrial structure in podocytes. Furthermore, Mitochondria in MTX2-KD podocytes were fragmented and punctate around the nucleus, while mitochondria in controls were uniformly distributed in tubes (Figure S7A). And overexpression of MTX2 had no influence on mitochondrial dynamics (Figure S7B). Therefore, mitochondrial dynamics and quality control network were altered. Mitochondrial outer membrane fusion protein mitofusin-2 (MFN2) and inner membrane fusion proteins optic atrophy1 (OPA1, including OPA1-L and OPA1-S) were reduced, whereas the levels of mitochondrial fission associated proteins dynamin-related protein 1 (DRP1) and fission protein 1 (FIS1) were increased, partly explaining the fragmented mitochondria in MTX2-KD podocytes (Figure 6D). Additionally, increased autophagosomes were observed in MTX2-KD podocytes, which verified with significantly increased LC3-II/I ratio, to alleviate the toxicity of fragment and dysfunction mitochondria (Figure 6E). Thus, these findings indicated that MTX2 deficiency caused abnormal mitochondrial structure through Sam50-CHCHD3-Mitofilin axis.

## Discussion

MTX2 was reported to be associated with MDPS with renal injury 22. In this study we identified three novel *MTX2* mutations in two children associated with proteinuria and kidney injury, genotyped as compound heterozygote and homozygote respectively. A series of podocyte and glomerular abnormalities were observed in Pod-Mtx2-KO mice from 8 weeks to old age, including microalbuminuria, glomerular mesangial hyperplasia, fusion and disordered arrangement of foot process, which replicated the renal clinical phenotype of children with MTX2 mutations. MTX2 is required for the maintenance of podocyte functions. Podocytes without MTX2 *in vitro* presented reductions of adhesion, migration and endocytosis, which were further restored by overexpression of MTX2. Moreover, MTX2 deficiency led to abnormal mitochondrial structure and dysfunction, including defects of complex I and III, increased ROS and decreased levels of Sam50-CHCHD3-Mitofilin axis in MIB complex for maintaining of cristae morphology.

Podocytes play an important role in the functional integrity of glomerulus. Reduced adhesive capacity leads to podocyte detachment from the GBM 24. Podocytes with impaired migration ability cannot fill up immediately to the site of podocyte shedding, thereby failing to maintain the stability of the GFB 25. Additionally, impaired endocytosis leads to abnormal turnover of SD components thereby resulting in SD injury and proteinuria 26,27. The exposed GBM lacking of podocytes coverage can adhere to the Bowman's capsule thus resulting in the occurrence of FSGS 3,28.

Mitochondrial dysfunction is associated with renal lesion or/and CKD 4,7,29. Here, MTX2 deficiency led to decreased enzymes activities of oxidative respiratory chain complex I and complex III, reduced OCRs and decreased ATP production, which in turn contributed to dysfunction of podocytes with decreased levels of adhesion, migration and endocytosis. In addition, decreased MMP was detected in MTX2-KD podocytes, implying that MTX2 deficiency resulted in reduced hydrogen ion pumping across the mitochondrial inner membrane and increased electron leakage during oxidative phosphorylation and electron transport as elsewhere 30,31, which led to dramatically raised ROS in podocytes. Mukhopadhyay et al. 32 reported that overproduced ROS played an important role in the pathogenesis of CKD. High concentrations of ROS cause mtDNA breaks, increase the risk of mtDNA mutations in offspring, decrease the efficiency of OXPHOS and damage lipids and proteins in kidney disease 33,34. Furthermore, elevated ROS and decreased MMP may accelerate apoptosis 32, which is consistent with increased apoptosis in MTX2-KD podocytes. The mitochondrial membrane regulates mitochondrial morphology, distribution, and function through a coordinated cycle of fission and fusion events, termed "mitochondrial dynamics" 35. In the present study, MTX2 deficiency caused abnormal mitochondrial dynamics with increased mitochondrial fission in podocytes, and accompanied with changes in mitochondrial ultrastructure. Mitochondrial swelling and vacuolization, cristae fragmentation and disorganization, and increased autophagosomes were observed in Pod-Mtx2-KO mice and MTX2-KD podocytes. These results illustrated that MTX2 deficiency led to mitochondrial dysfunction and abnormal morphological structure, which further induced podocyte injury. Increased autophagy may be associated with phagocytosis and clearance of damaged mitochondria, so as to protect MTX2-KD podocytes from injury 36.

Most studies have shown that mitochondrial cristae play a critical role in the occurrence of mitochondria initiated intrinsic apoptosis triggered by cytochrome c release 37,38. Upon stimulated by apoptotic signals, mitochondria undergo extensive inner membrane remodeling, including changes in cristae morphology as well as the expansion of cristae junctions, resulting in dissociation of Cyt c into the mitochondrial intermembrane space. Subsequently Cyt c is released into the cell matrix across the outer membrane, stimulating the delivery of the next apoptotic signal 38,39. In the present study, MTX2 deficiency resulted in increased apoptosis in podocytes. Furthermore, the protein levels of pro-apoptotic factors including Cyt c, Bim, Bax, bad, bid, caspase-3 and caspase-8 were significantly up-regulated in MTX2-KD podocytes by apoptotic protein microarray assay. Combined with the disorganized mitochondrial cristae arrangement in podocytes with MTX2 deficiency, it can be concluded that MTX2 deficiency led to structure abnormalities of mitochondrial cristae in podocytes, which further induced the occurrence of apoptosis.

Mitochondrial cristae are also crucial for the proper function of mitochondria as a whole, anchoring a series of mitochondrial respiratory chain supercomplexes responsible for oxidative phosphorylation, and serving as an important site for ATP production 40,41. Abnormal cristae structure can lead to mitochondrial dysfunction and thus affect the proper function of the entire cell. However, how the architecture of cristae is precisely organized remains largely unclear. In the present study, the protein levels of Sam50, CHCHD3 and Mitofilin as well as MTX1were decreased in MTX2-KD podocytes. No significant change was observed in transcripts of either Sam50, CHCHD3 nor Mitofilin between MTX2-KD and control podocytes (Figure S8). There were also undetected notable impacts of nonsense-mediated mRNA decay on protein expression as UPF1 was comparable in both MTX2-KD and control cells (Figure S9) 42. Obviously, the reduced MTX2 led to the instability of Sam50-CHCHD3-Mitofilin axis, which primarily mediates the connection between SAM and MICOS complexes to assemble MIB supercomplex for maintaining mitochondrial cristae morphology. Tang, et al. 43 have reported that OMA1-mediated cleavage of CHCHD3 leads to disruption of Sam50-CHCHD3-Mitofilin axis, which detaches SAM and MICOS complexes, resulting in MIB disassembly and consequently abnormal mitochondrial cristae architecture. Combining significant depletion of all three protein levels in the Sam50-CHCHD3-Mitofilin axis and disorganized mitochondrial cristae in podocytes with MTX2 deficiency, it can be concluded that MTX2 deficiency led to defects in the Sam50-CHCHD3-Mitofilin axis, which further induced abnormal mitochondrial cristae structure and consequent mitochondrial dysfunction, ultimately leading to podocyte damage and proteinuria. Our finding here provides new insights into the pathophysiology of glomerular disease associated with mitochondrial dysfunction.

The limitations of this study are as following: 1) Although the study displays that the MTX2 deficiency resulted in other MIB complex components dysregulation, its underlying reason is still unclear. 2) We focus MTX2 function on podocyte here as patients with *MTX2* mutations manifested proteinuria and glomerulopathy. However, its function on renal tubular cells requires further investigation.

## Methods

### Subjects

Proband 1 was ascertained at Wuhan women and children's health care center, Wuhan, China. Proband 2 was diagnosed at Xi'an Children's Hospital, Xi'an, China. After informed consent were obtained from family members, blood samples from probands and their parents were taken for extraction of genomic DNA, and some family members were interviewed for clinical manifestations and personal medical history. The probands underwent blood and urine laboratory tests, B-ultrasound, CT, ECG, EEG and renal pathology during hospitalization.

### Generation of conditional podocyte-specific Mtx2 knockout mice

Podocin-Cre mice were crossed with Mtx2 ^flox/flox^ mice to obtain Mtx2^ flox/+^ Cre (+) mice. Then, Mtx2^ flox/+^ Cre (+) mice were crossed with Mtx2 ^flox/flox^ mice to generate Mtx2^ flox/flox^ Cre (+) mice (Pod-Mtx2-KO mice), which were identified by tail genotyping (PCR) with primers: Podocin-cre Forward primer: CGGTTATTCAACTTGCACCA; Podocin-cre Reverse primer: GCGCTGCTGCTCCAG; Mtx2 Forward primer: CCAGTGGGCACTTGGATATAGAC; Mtx2 Reverse primer: TGCCAAAGCATTATCCAGTTATCC. Mtx2^ +/+^ Cre (+) and Mtx2 ^flox/flox^ Cre (-) mice were used as controls 44.

### Urinary albumin measurements

Random urine was collected from Pod-Mtx2-KO and control mice, and urinary protein was initially qualitatively assessed by Coomassie blue staining. The 24 h urine of mice was subsequently collected with metabolism cages and the 24 h urine volume was counted, the urinary albumin concentration was measured using Mouse Albumin Detection Kit (Chondrex, Woodinville, USA) following the manufacturer's instructions, then 24 h urinary albumin quantification was calculated.

### Isolation and culture of primary glomerular podocytes

Mice were sacrificed after anesthesia, removed kidneys were placed in prechilled PBS, renal envelopes were peeled off, and renal cortices were rapidly cut into small sections and placed in a 70-μm cell strainer (Falcon; BD Biosciences, USA) for grinding. The strainer was subsequently rinsed with PBS, and the filtrate were sieved through a 40 μm cell strainer (Falcon; BD Biosciences, USA). Then the glomeruli remaining on the strainer were collected by PBS and seeded into culture flasks. The remaining tubules and their surrounding cells were scraped off on day 5. On day 6-7, digested primary glomerular cells were screened through a 40 μm cell strainer then seed on a new culture dish. On day 11-12, primary glomerular podocytes can be used for the experiment 45.

### Immunohistochemistry and immunofluorescence staining

Sections of renal tissue (3-5 μm) were fixed with 4% paraformaldehyde (PFA) for 48 h at room temperature (RT) and subsequently performed by the Morphohistology platform, Zhejiang University School of Medicine for dehydration, paraffin imbedding, then processed for HE staining and Masson staining. Images were taken by a Leica fluorescence orthotopic microscope (Leica DM4000, Germany).

For immunofluorescence, frozen renal sections (4-5 µm) and podocytes on the glass slides were fixed with 4% PFA overnight at 4 °C, permeabilized with 0.3% Triton X-100 for 30 min, and blocked in 5% bovine serum albumin (BSA) for 1 hour at RT, then incubated with primary antibody solutions overnight at 4 °C followed by incubation with secondary antibody for 1 h in the dark at RT. Images were taken by a Leica sp8 laser (TCS SP8, Germany) scanning confocal microscope.

### Mitochondrial fluorescence labeling

Mature podocytes were incubated with mitotracker (Beyotime, CN) staining solution for 30 min in the dark at RT and fixed with 4% PFA overnight at 4 °C, then incubated with DAPI staining solution for 5 min in the dark at RT. Images were taken by a Leica sp8 laser scanning confocal microscope.

### Transmission and scanning electron microscopy

The 1×1×1 mm kidney cortex and podocyte pellets were fixed with 2.5% glutaraldehyde at 4 °C overnight. For TEM, samples were fixed with 1% osmic acid for 1-1.5 h, 2% uranyl acetate for 30 min, dehydrated sequentially with 50%, 70% and 90% ethanol for 15 min each, 100% ethanol for 20 min and 100% acetone twice for 20 min each, then processed for embedding, polymerization, staining, and localization. For scanning EM, samples were fixed with 1% osmic acid for 1-1.5 h and dehydrated sequentially with 50%, 70%, 90% ethanol for 15 min each and with 100% ethanol twice 20 min each, then processed for critical point drying, coating. Sample processing and detection were performed by the Center of Cryo-Electron Microscopy, Zhejiang University.

### MPC5 culture and transfection

MPC5 is a gift from Professor Peter Mundel (Mount Sinai School of Medicine, New York, USA). MPC5 in a proliferative state were cultured at 33 °C in RPMI medium containing 10% fetal bovine serum (FBS) and 10 U/ml mouse recombinant interferon-γ (R&D Systems, Minneapolis, MN). MPC5 were subsequently passaged to RPMI medium containing 10% FBS in the absence of interferon-γ to induce differentiation at 37 °C for 10-14 d. Lentivirus system (GenePharma, Shanghai, CN) was used to constructed MTX2 deficient or overexpressing cells. MPC5 was seeded into 6-well plates at a density of 5×10^5^ cells and cultured at 33 °C overnight. Subsequently an appropriate volume (100 μl) of lentivirus was added to each well. After incubation at 33 °C for 24 h, cells were incubated with fresh medium, and then selected with 2.5 μg/ml puromycin for 7 d to achieve a transfection efficiency >95%. ShRNA sequences for knockdown of Mtx2: GGGCAAATGCGGAATATATGT.

### Podocyte function assays

For adherence analysis, 1 ml RPMI medium and 1 ml cell suspension at a density of 4×10^5^/ml were added to 6-well plates coated with collagen, followed by incubation at 37 °C for 1 h, then fixed with 4% PFA. Images were taken by Leica DM4000 (Germany) for statistics analysis. For abscission assess, cells at 80% confluence density were stimulated with 100 μg/ml puromycin aminonucleoside at 37 °C for 48 h, then counted the adherent cells under Leica DM4000.

For migration analysis, 8-μm Transwell kit (Falcon) coated with collagen were hanged on 24-well plates containing 500 μl RPMI medium with 10% FBS. 100 μl cell suspension at a density of 2×10^5^/ml were added to the upper layer of the chamber, followed by incubation at 37 °C for 24 h. Cells from the bottom side of the chamber (migrated cells) were fixed with 4% PFA and stained with 1% crystal violet solution. Migrated cells were observed and counted by Leica DM4000 (Germany).

For endocytosis analysis, MPC5 was cultured in PBS containing FITC-transferrin (50 µg/ml) at 37 °C for 5 h. After washed with PBS and citrate buffer, cells were fixed with 4% PFA and stained with DAPI. Images were taken by a Leica sp8 laser (TCS SP8, Germany) scanning confocal microscope. The endocytosis abilities were indicated by the mean fluorescence intensity of FITC-transferrin.

### Western blotting

Podocytes were lysed by RIPA (#89901; Thermo Fisher) containing a protease inhibitor cocktail (ab65621; Abcam, dilution 1:200) to extract total proteins. Then the protein concentration was detected with a Pierce BCA Protein Assay kit (#23225; Thermo Fisher). Extracted total proteins were denatured at 96 °C for 5 min, electrophoresed on 10% sodium dodecyl sulfate-polyacrylamide (SDS-PAGE) gels, subsequently transferred to polyvinylidene difluoride (PVDF) membranes. Membranes were blocked in 5% BSA for 1 h at room temperature, then incubated with primary antibody solutions overnight at 4 °C followed by incubation with HRP-conjugated secondary antibody for 1 h at RT. Signals were detected with enhanced chemiluminescence reagents (#32209; Thermo Fisher).

Antibodies used in this work were as follows: MTX2 (11610-1-AP, Proteintech) diluted at 1:250 for immunohistochemistry (IHC) and 1:600 for western blotting (WB); MTX2 (sc-514231, Santa Cruz) diluted at 1:250 for immunofluorescence (IF); synaptopodin (21064-1-AP, Proteintech) diluted at 1:400 for IF; Alexa Fluor 594 donkey anti-rabbit IgG (H+L) secondary antibody (A21207; Life Technologies) diluted at 1:500 for IF; Alexa Fluor 488 Goat anti-mouse IgG, IgM (H+L) secondary antibody (A-10680, Invitrogen) diluted at 1:10000 for IF; WT1 (DF6331, Affinity Biosciences) diluted at 1:250 for IF; ND2 (A17968, ABclonal) diluted at 1:1000 for WB; ND4 (A9941, ABclonal) diluted at 1:1000 for WB; ND5 (A17972, ABclonal) diluted at 1:1000 for WB; CYTB (A17966, ABclonal) diluted at 1:1000 for WB; MTCO2 (55070-1-AP, Proteintech) diluted at 1:2000 for WB; NDUFA9 (A3196, ABclonal) diluted at 1:1000 for WB; NDUFS1 (12444-1-AP, Proteintech) diluted at 1:5000 for WB; SDHB (10620-1-AP, Proteintech) diluted at 1: 20000 for WB; UQCRC1 (21705-1-AP, Proteintech) diluted at 1:4000 for WB; ATPB (17247-1-AP, Proteintech) diluted at 1:3000 for WB; TOM20 (A19403, ABclonal) diluted at 1:5000 for WB; MFN2 (12186-1-AP, Proteintech) diluted at 1: 5000 for WB; OPA1 (27733-1-AP, Proteintech) diluted at 1:2000 for WB; DRP1 (12957-1-AP, Proteintech) diluted at 1:5000 for WB; FIS1 (10956-1-AP, Proteintech) diluted at 1:1500 for WB; LC3B (ab192890, Abcam) diluted at 1:2000 for WB; Metaxin 1 (sc-135989, Santa Cruz) diluted at 1:500 for WB; VDAC1/2 (10866-1-AP, Proteintech) diluted at 1:1500 for WB; TOMM40 (18409-1-AP, Proteintech) diluted at 1:8000 for WB; Sam50 (28679-1-AP, Proteintech) diluted at 1:8000 for WB; Mitofilin (10179-1-AP, Proteintech) diluted at 1:1000 for WB; CHCHD3 (25625-1-AP, Proteintech) diluted at 1:8000 for WB.

### Cell apoptosis assay

Podocytes were digested with trypsin without EDTA, and resuspended with 1×Binding buffer to adjust cell density to 1×10^6^ /ml. 100 μl cell suspension was subsequently added into the tube and mixed with 5 μl propidium iodide and 5 μl Annexin V (#556547, BD Biosciences, USA) followed by incubation in the dark for 15 min at RT. Then 1×Binding buffer was added into the tube to make the final volume of the liquid to be 500 μl and apoptosis was measured by CytoFLEX LX (Beckmancoulter, USA).

### Apoptotic protein microarray

To clarify the mechanism of podocyte apoptosis, podocytes from MTX2-KD and control group were lysed by RIPA (#89901, Thermo Fisher) to extract total proteins. The Mouse Apoptosis Antibody Array (#AAH-APO-1, RayBiotech, Norcross, GA, USA) was used to detect changes in the protein levels of 38 indicators of mouse apoptosis according to the manufacturer's procedures. The spots were scanned and analysed using a chemiluminescence imaging analysis system (ImageQuant LAS4000, GE Healthcare Corporate, USA).

### Enzyme activities of oxidative respiratory chain complexes

Mitochondrial homogenates were prepared on ice, and protein concentrations of homogenates were determined by the Total protein quantitative assay kit (A045-2-2, Nanjing Jiancheng Bioengineering Institute). Following the manufacturer's instructions, the enzyme activities of oxidative respiratory chain complexes (complex I-V) were measured using the Electron transport chain Complex assay kit (A089-1-1, A089-2-1, A089-3-1, A089-4-1, A089-5-1, Nanjing Jiancheng Bioengineering Institute).

### Mitochondrial membrane potential assay

According to the manufacturer's instructions, MMP was detected using a Mitochondrial membrane potential assay kit with JC-1 (C2006, Beyotime, CN). Briefly, 1×10^5^-6×10^5^ podocytes were collected and resuspended with 0.5 ml JC-1 staining solution and 0.5 ml RPMI medium, then incubated in the dark for 20 min at 37 °C and detected by CytoFLEX LX (Beckmancoulter, USA). The level of mitochondrial membrane potential was indicated by the ratio of the mean fluorescence intensity of JC-1 multimers and monomers.

### Oxygen consumption rate

Mitochondrial OCR was detected by XF^e^96 extracellular flux analyzer (Seahorse Bioscience, North Billerica, USA) using seahorse XF Cell mitochondrial stress test kit (#103015-100, Agilent) following the manufacturer's instructions, as detailed elsewhere 46. MPC5 were seeded into XF96 special cell culture plates (#101085-004, Agilent) coated with collagen at a density of 2×10^4^ cells per well and cultured at 37 °C overnight. The OCR was detected for each well throughout the procedural injections: Oligomycin (1.5 μM), FCCP (2 μM) and Retenone /Antimycin (0.5 μM).

### ATP measurement

The podocytes ATP production was measured using the Enhanced ATP Assay Kit (S0027, Beyotime, CN) according to the manufacturer's procedures.

### Mitochondrial ROS assay

The mitochondrial ROS was detected using the ROS Assay Kit (S0033S, Beyotime, CN) following the manufacturer's instructions. Briefly, 2×10^6^ podocytes were collected and resuspended with 1 ml RPMI medium and 1 μl DCFH-DA solution, then incubated in the dark for 20 min at 37 °C. The fluorescence intensity was measured by CytoFLEX LX (Beckmancoulter, USA) with an emission at 525 nm and excitation at 488 nm.

### Statistical analysis

Results were statistically analyzed using GraphPad Prism (Version 8.0) and presented as the mean ± SD. Comparisons between two groups were performed using Two-tailed Student's test.* P* < 0.05 was considered to be statistically significant.

## Supplementary Material

Supplementary figures and tables.
