# Supplementary material for: Loss of MTX2 causes mitochondrial dysfunction, podocyte injury, nephrotic proteinuria and glomerulopathy in mice and patients
Source: Int J Biol Sci. 2024 Jan 12;20(3):937–52. doi: 10.7150/ijbs.89916 (PMC10797693; doi:10.7150/ijbs.89916)
Supplement: Supplementary file 1 — Supplementary figures and tables. [file ijbsv20p0937s1.pdf]

Supplementary figure 1

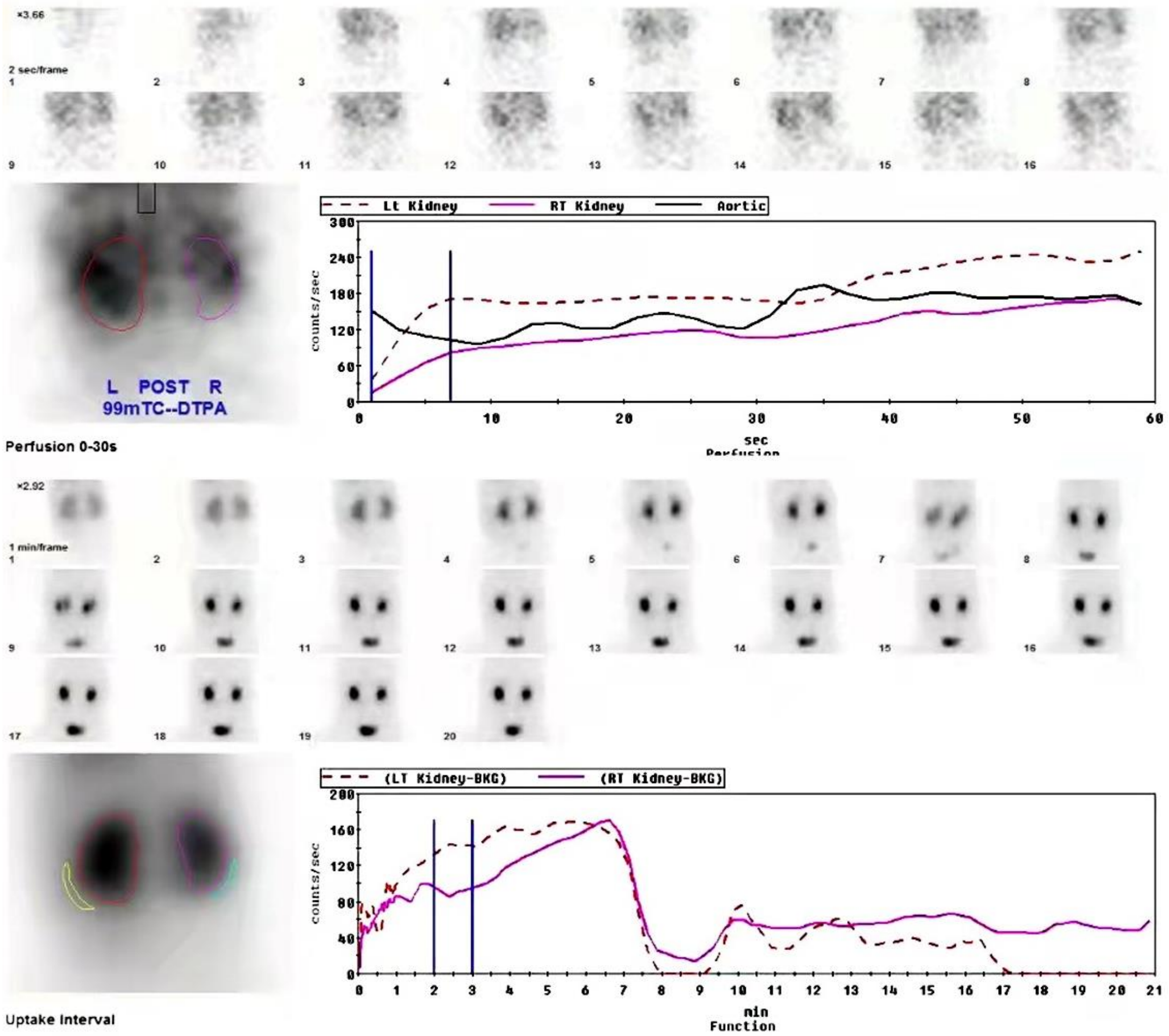

GFR ml/min: 66.71

Kidney	Left	Right
Kidney Area (cm2):	24.98	16.4
Kidney depth (cm):	3.	3.
Perfusion% (Int):	70.07	29.93
Perfusion% (Slo):	86.56	13.44
Uptake% (Int):	60.77	39.23
GFR:	40.54	26.17

Kidney	Left	Right
Time to peak:	5.61	6.51
Peak to 1/2 peak:	1.75	.75
20min/peak ratio:	NA	.3
20min/3min ratio:	NA	.57

Figure S1. Renal dynamic imaging in proband 1.
Renal dynamic imaging demonstrated mildly decreased blood perfusion and glomerular filtration in the right kidney and bilateral upper urinary tract excretory obstruction.

Supplementary figure 2

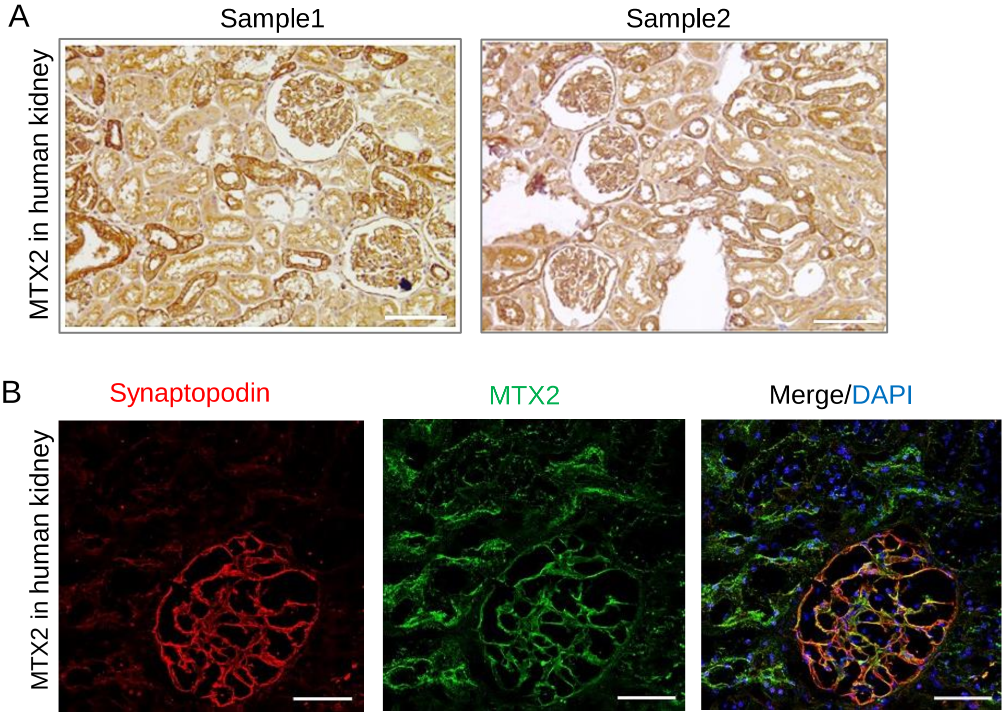

Figure S2. Expression of MTX2 in kidneys of human.
(A) The expression of MTX2 in sections of human kidney by immunohistochemistry. Scale bar: 50 μm. (**B)** The expression of MTX2 in podocytes in human kidney by immunofluorescence. scale bars: 40 μm. Samples were collected from patients with isolated hematuria.

Supplementary figure 3

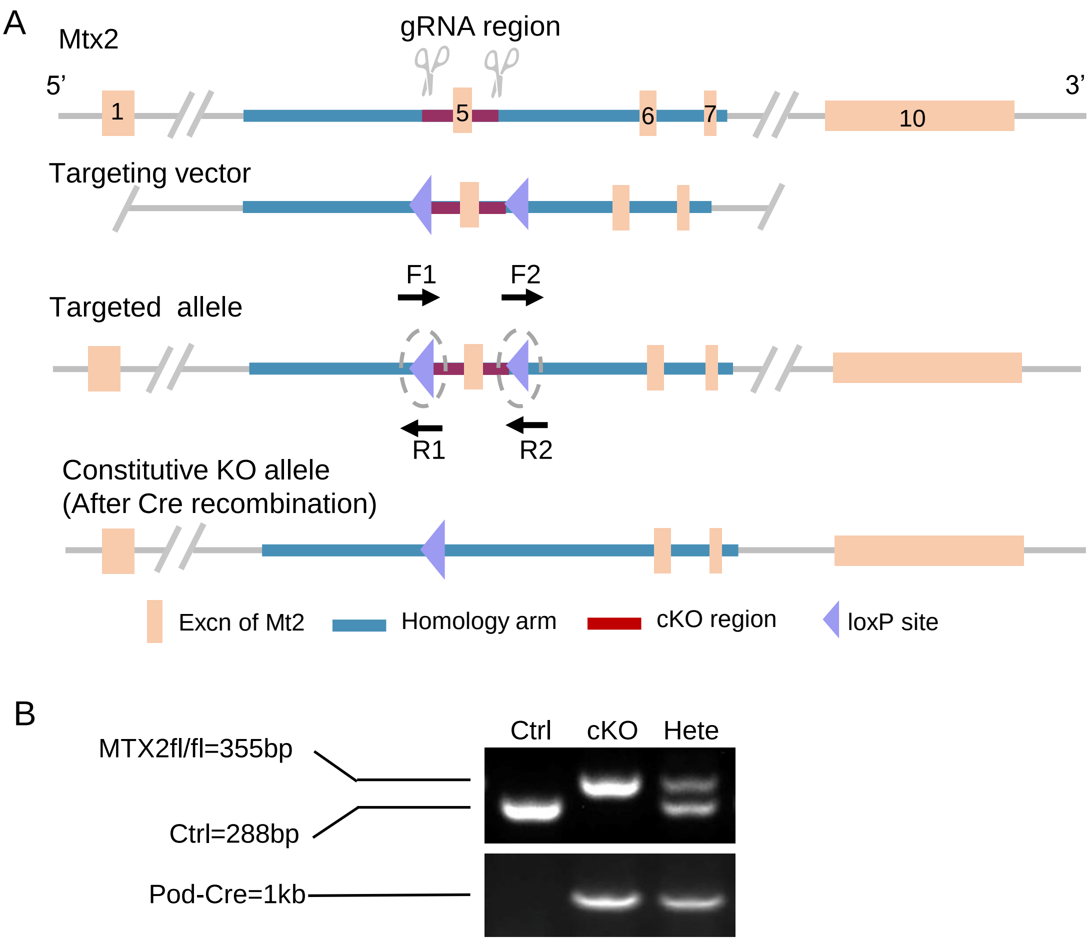

Figure S3. Construction of Pod-Mtx2-KO mice using the Cre-Loxp recombination system. **(A)** Schematic diagram for Pod-Mtx2-KO mice. **(B)** Genotyping of mice at 4 weeks of age by PCR. Ctrl, (Mtx2 +/+ Cre (-), DNA,288bp); cKO, Pod-Mtx2-KO mice, (Mtx2 flox/flox Cre (+), DNA, 355bp & 1kb); Hete, heterozygote, (Mtx2 flox/+ Cre (+), DNA, 355bp & 288bp & 1kb). Mtx2 Forward primer 1: CCAGTGGGCACTTGGATATAGAC; Mtx2 Reverse primer 1: TGCCAAAGCATTATCCAGTTATCC. Podocin-cre Forward primer1: CGGTTATTCAACTTGCACCA; Podocin-cre Reverse primer 1: GCGCTGCTGCTCCAG.

Supplementary figure 4

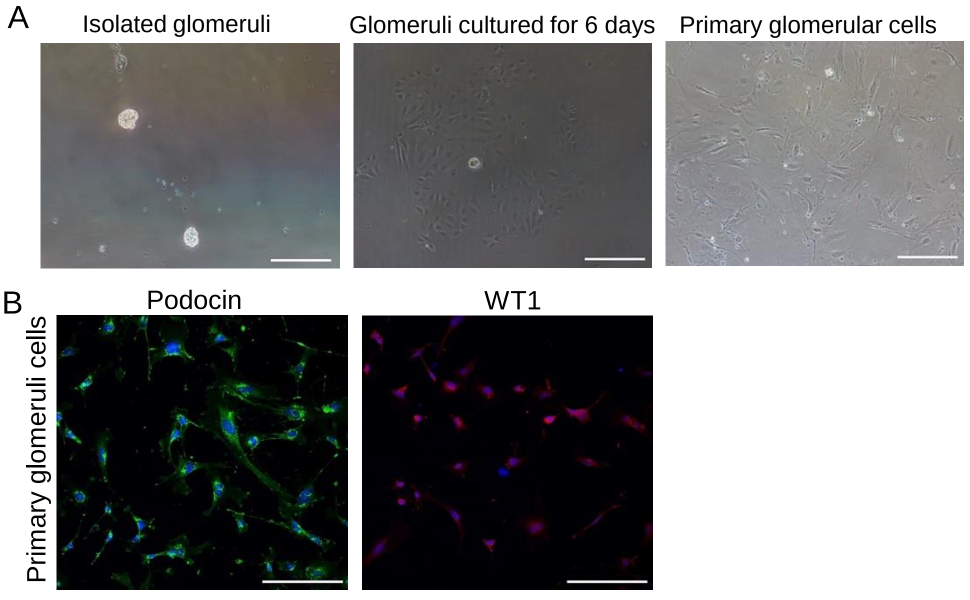

Figure S4. Culture and identification of primary glomerular cells.
(A) Isolation and culture of primary glomerular cells from 6-week mice. Bar: 250 μm .
(B) Identification of glomerular cells with antibodies podocin and WT1. Podocin & WT1, markers of podocytes. Bar: 160 μm.

Supplementary figure 5

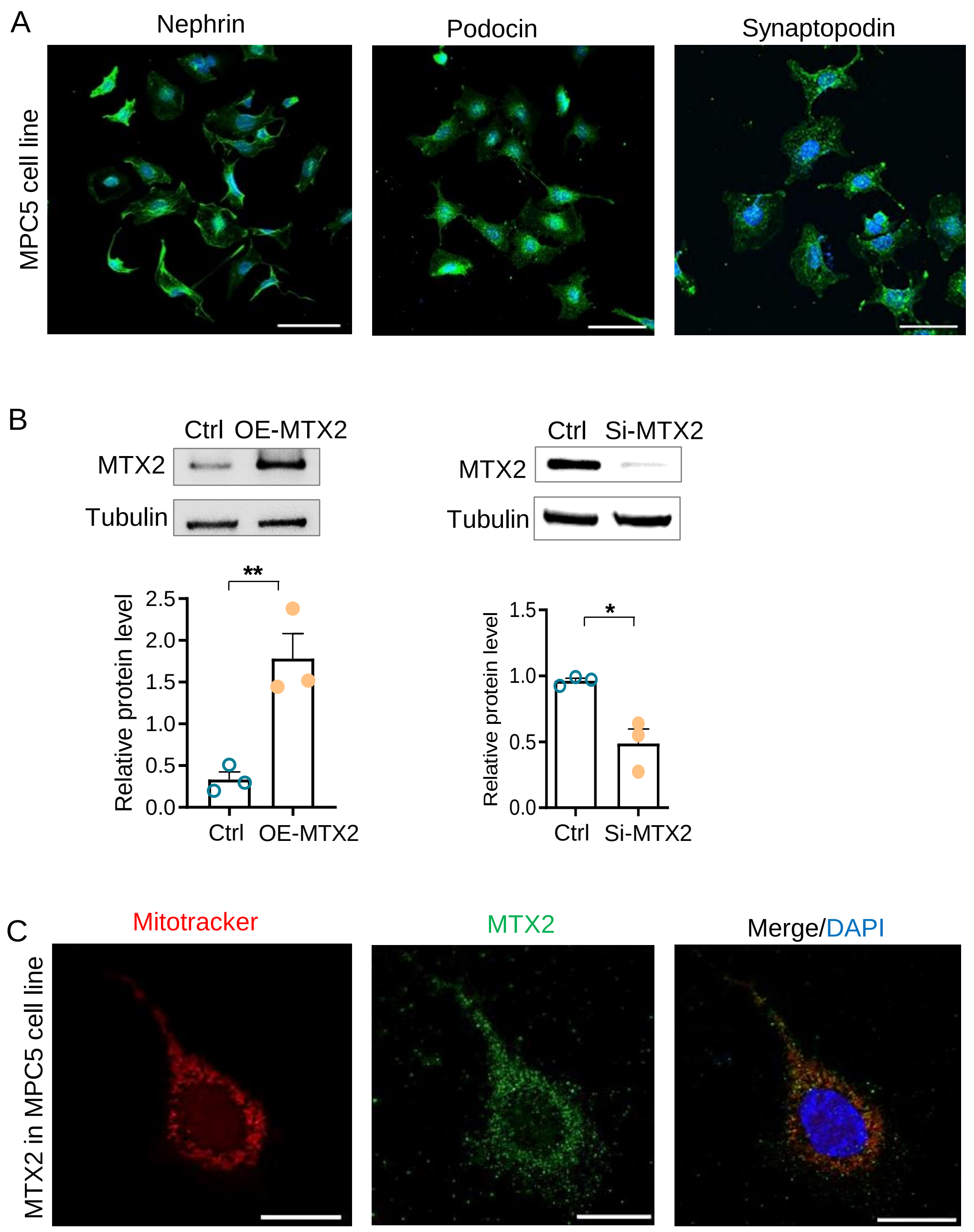

Figure S5. MPC5 cell line and MTX2 expression and localization in MPC5.
(A) Identification of MPC5 cells with antibodies nephrin, podocin and synaptopodin. Bar: 60 μm.
(**B**) MTX2 knockdown or overexpression by lentiviral transfection in MPC5.
(C) MTX2 co-localized with mitochondria. Bar: 15 μm.

Supplementary figure 6

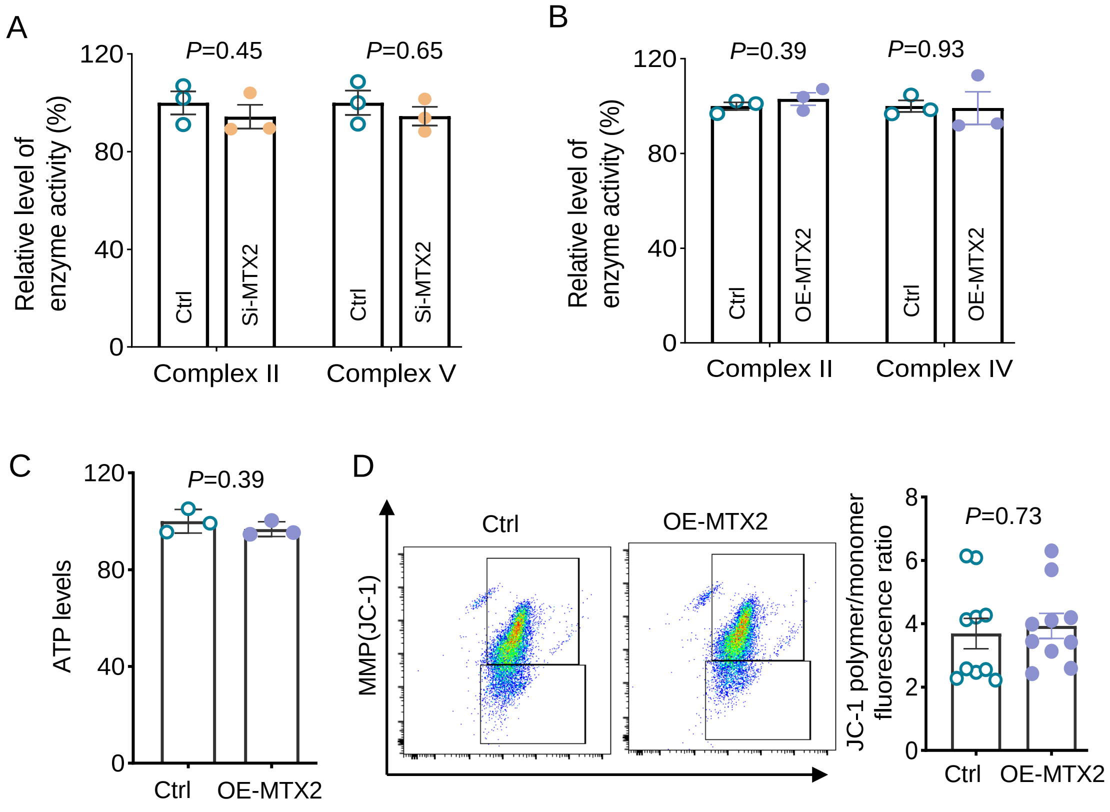

Figure S6. Mitochondrial function in MPC5 with or without MTX2.
(A) Deficiency of MTX2 had no influence on activity of mitochondrial complex II or complex V.
(B) Overexpression of MTX2 had no influence on activity of mitochondrial complex II or complex IV.
(C) Whole cell ATP production in MPC5 with MTX2 overexpression.
(D) Mitochondrial membrane potential in MPC5 with MTX2 overexpression.

Supplementary figure 7

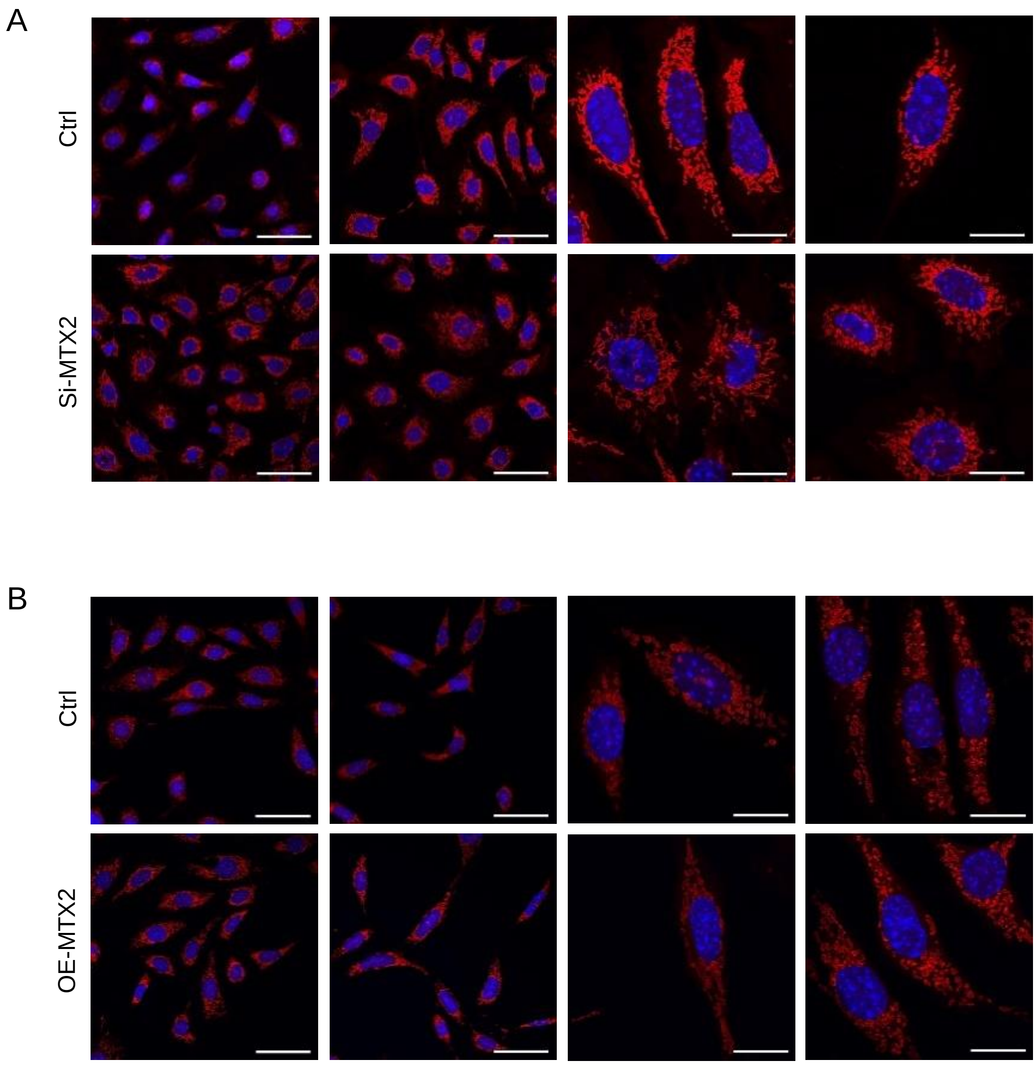

Figure S7. Mitochondrial dynamics in MPC5 with or without MTX2.
(A) Representative images of mitochondrial immunofluorescence staining by mitotracker showing fragmented and punctate mitochondria around the nucleus in MTX2-KD podocytes, uniformly distributed mitochondria in tubes in controls. Bar: 40 μm (columns 1 and 2), 15 μm (columns 3 and 4) (n = 3).
(B) Representative images demonstrating evenly distributed mitochondria in tubes in both MTX2 overexpressing and control podocytes. Bar: 40 μm (columns 1 and 2), 15 μm (columns 3 and 4) (n = 3).

Supplementary figure 8

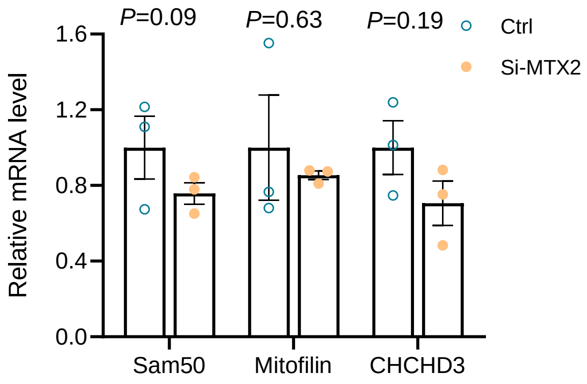

Figure S8. The mRNA levels of Sam50, Mitofilin and CHCHD3 in MPC5 with or without MTX2.

Supplementary figure 9

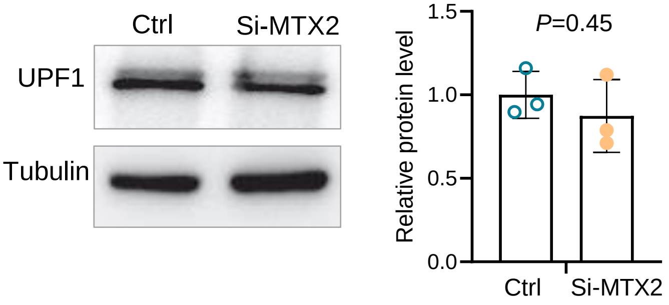

Figure S9. Western blots analysis of UPF1 in MTX2-KD and control podocytes.

Table S1. 24 h urinary albumin quantification of Pod-Mtx2-KO and control mice at 8, 12, 16 weeks of age

Weeks	Urinary albumin (μg/24 h)	
	Ctrl	cKO
8 W	16.35±1.289	20.28±0.6441*
12 W	16.24±0.6059	21.22±0.4362***
16 W	16.23±1.763	21.5±0.3214***

cKO: conditional podocyte-specific Mtx2 knockout mice; *, $P < 0.05$, ***, $P < 0.0001$.

Table S2. Up-regulated pro apoptotic factors in MTX2-KD podocytes by mouse apoptotic protein microarray assay.

Protein ID	Protein description	Fold change (MTX2 knockdown vs control)	Regulation
BIM	Bcl-2 interactingmediator of cell death	2.379056546	up
BAX	BCL2 associated X, apoptosis regulator	1.877139223	up
BAD	BCL2 associated agonist of cell death	1.722309417	up
BID	BH3 interacting domain death agonist	2.410412061	up
Capase-3	Cysteinyl aspartate specific proteinase 3	2.335875155	up
Capase-8	Cysteinyl aspartate specific proteinase 8	123.2492439	up
CytoC	cytochrome c	2.374809122	up
IGFBP-5	Insulin Like Growth Factor Binding Protein 5	2.386988077	up
IGFBP-3	Insulin Like Growth Factor Binding Protein 3	1.569901317	up
HSP70	Heat shock protein 70	2.979142851	up
p53	Tumor Protein P53	1.803953355	up
p27	Tumor Protein P27	1.754715968	up
p21	Tumor Protein P21	1.53034612	up
DR6	Death Receptor 6	4.533755208	up
HTRA2	HtrA serine peptidase 2	3.083498106	up
SMAC	Diablo IAP-binding mitochondrial protein	6.613224268	up
